# Parental educational level and injury incidence and mortality among foreign-born children: a cohort study with 46 years follow-up

**DOI:** 10.5249/jivr.v6i1.525

**Published:** 2014-01

**Authors:** Omid Beiki, Najmeh Karimi, Reza Mohammadi

**Affiliations:** ^*a*^Research Center for Environmental Determinants of Health, Kermanshah University of Medical Sciences, Kermanshah, Iran.; ^*b*^Unit of Clinical Epidemiology, Department of Medicine Solna, Karolinska Institutet, Stockholm, Sweden.; ^*c*^Department of Public Health, Karolinska Institutet, Stockholm, Sweden.

**Keywords:** Unintentional injury, Educational level, Relative risk, Cohort study

## Abstract

**Background:**

Injury risk during childhood and adolescence vary depending on socio-economic factors. The aim of this study was to study if the risk of fatal and non-fatal unintentional injuries among foreign-born children was similar across parental educational level or not.

**Methods:**

In this retrospective cohort study we followed 907,335 children between 1961 and 2007 in Sweden. We established the cohort by linkage between Swedish national registers including cause of death register and in-patient register, through unique Personal Identification Numbers. The main exposure variable was parental (maternal and paternal) educational level. The cohorts was followed from start date of follow-up period, or date of birth whichever occurred last, until exit date from the cohort, which was date of hospitalization or death due to unintentional injury, first emigration, death due to other causes than injury or end of follow-up, whichever came first. We calculated hazard ratios (HR) with 95% confidence intervals (95% CI) by Cox proportional hazards regression models.

**Results:**

Overall, we found 705 and 78,182 cases of death and hospitalization due to unintentional injuries, respectively. Risk of death and hospitalization due to unintentional injuries was statistically significantly 1.48 (95% CI: 1.24-1.78) and 1.10 (95% CI: 1.08-1.12) times higher among children with lowest parental educational level (9 years and shorter years of study) compared to children with highest parental educational level (+13 years of study). We found similar results when stratified our study group by sex of children, by maternal and paternal educational level separately, and injury type (traffic-related, fall, poisoning, burn and drowning).

**Conclusions:**

It seems injury prevention work against unintentional injuries is less effective among children with low parental education compared with those with higher parental education. We recommend designing specific preventive interventions aiming at children with low parental education.

## Introduction

Intentional and unintentional injuries are the main causes of child and adolescent morbidity and mortality in the world.^1,2^ According to World Health Organization (WHO), UNICEF and Euro Safe, injury is the leading cause of death in children and adolescents younger than 19 years.^3,4^

Children are exposed to a wide variety of unintentional injuries including traffic crashes, drowning, burns, falls, poisoning and others. There are growing evidence that lower socio-economic status (SES) is associated with injury morbidity and mortality among children.^5-9^ Because the process of selecting the best SES indicator is difficult, the SES indicator selected varied from study to study. Regardless of feasibility of performing migrant studies only a limited number of studies in this kind have been published. Although many studies have been published on injury among foreign-born children, however, only a few number of them take parental education into consideration. Those studies that aimed at examination of the effect of parental education on injury, also, were hampered by lack of power and were not able to comprehensively report results by type of injury in details.

Migration is a major component of population change and immigration has been increasing in Sweden over recent decades and accounts for up to 20% of all young people in the country. These migrants include foreign-born persons together with the Sweden-born persons with two foreign-born parents and different nationalities represented within this group of immigrants in Sweden.^10^ Migration, involves a series of events that can place migrants and their families at risk. Migrants’ health is associated with many factors that influence their health. They are more vulnerable to different types of injuries, for example immigrants’ children 5 to 9 years old have more traffic and other crashes than native children or in other country poisoning and burn is higher among immigrants than native children.^11,12^

The aim of this study is to compare the risk of fatal and non-fatal unintentional injuries among children with lowest parental education with that among children with highest parental education.

## Methods

**Study population**

According to the United Nations “Convention on the Rights of the Child” a child is defined as any human being below the age of 18.^13^ The study population consisted of all children born in Sweden during the period 1961-2007. All individuals registered in Sweden are given a Personal Identity Number (PIN) as identification. We created a database by linkages using PIN between The National Population and Housing Censuses, Swedish Total Population Register (TPR), Cause of Death Register, National Patient Register (NPR), Multi-Generation Register, Immigration Register, and Longitudinal Integration Database for Health Insurance and Labor Market (LISA). We found 907,335 foreign-born children who were born between 1961 and 2007. Among them, 388,085 (42.8%) were with both parents born outside Sweden, 265,854 (29.3%) with father foreign-born and mother Sweden-born, and 253,396 (27.9%) with mother foreign-born and father Sweden-born.

The Total Population Register is the basic register of the population of Sweden, and contains information about sex, age, marital status.^14,15^ Cause of Death Register (CDR) includes all deaths. The causes of death are classified according to the International Classification of Diseases (ICD).^16^ Multi-Generation Register contains connections between all persons born since 1932 and their biological parents and when data on country of birth is missing in the TPR, this information is taken from the Multi-Generation Register.^17^ The National Population and Housing Censuses cover demographic, occupational, and socioeconomic factors, such as income, occupation, and education for each household member. Parental education is obtained from this register. The Immigration Register contains data on immigration, emigration, and country of birth.^14^ In the 1964 the National Board of Health and Welfare started to collect information regarding in-patients at public hospitals, the National Patient Register (NPR). From 1987 NPR includes all in-patient care in Sweden. The information in NPR can be divided into four different groups. These groups consist of several variables: patient data, geographical data, administrative data and medical data.

**Variables**

The main exposure variable for study was parental education. To be comprehensive we stratified children with foreign-background (with at least one parent foreign-born) into three different groups; children with both parents foreign-born, children with only mother foreign-born, and children with only father foreign-born. 

The outcome variables were defined according to the different revisions of the WHO International Classification of Diseases-ICD.^18,19^ We used ICD-7 during 1958–68 and the 8th revision of the ICD during 1969–1986, ICD-9 during 1987–96 and the 10th revision of the ICD during 1997–2007. We extract events of hospitalizations due to unintentional injury from variable external cause of injury and poisoning in National Patient Register based on Swedish version of ICD. We extracted external causes of death from the Cause of Death Register in which international versions of ICD were used for registering data. 

**Statistical Analysis**

To compare the risk of fatal and non-fatal unintentional injuries among children with highest parental education with that among children with lowest parental education, multivariate analyses of risk were conducted using Cox proportional hazard regression models (SAS 9.23). We followed each child from his/her birth date (or start date of study; January 1st, 1961 for those born before this date) until emigration date, date of death or hospitalization due to injury, end of follow-up (or date of end of study; December 31st, 2007) or at date children turn to 18 years of age whichever occurred first. We calculated Hazard ratios (HR) with 95% confidence intervals (CIs) for injuries. To adjust for the probable effect of age and period effect, we put age at exit and calendar period of birth into the statistical models. A difference was considered statistically significant if P value was < 0.05. Point estimates and 95% confidence intervals were produced using the maximum partial likelihood for the effect estimates. The validity of the proportional hazards assumption was evaluated using a martingale residual-based graphical and numerical approach. ^20^ We stratified all statistical analyses by child sex.

**Ethical consideration**

To ensure confidentiality, the Personal Identity Numbers are replaced with serial numbers by Statistics Sweden. This study was approved by the Regional Board of The Ethical Committee, Stockholm.

## Results

Overall, we found 78,182 and 705 cases of hospitalization and death among foreign-background children due to unintentional injuries, respectively (Table 1-2).

**Table 1:** Hazard Ratio* (HR) and 95% confidence interval (95% CI) of non-fatal injuries among children 0-18 years old with foreign background by parental education, 1961-2007

Attained Parental Education	Male		Female		All	
	Event	HR (95% CI)	Event	HR (95% CI)	Event	HR (95% CI)
Unknown	279	1.04 (0.93-1.18)	185	1.00 (0.87-1.16)	464	1.03 (0.94-1.13)
<9 years	7105	**1.12 (1.09-1.16)**	4203	1.03 (0.99-1.06)	11308	**1.08 (1.06-1.11)**
10-12 years	23195	**1.11 (1.09-1.13)**	14875	**1.08 (1.06-1.11)**	38070	**1.10 (1.08-1.12)**
>=13 years	16998	1(reference)	11342	1(reference)	28340	1(reference)

*Adjusted for attained age and calendar period of birth. Bold type, 95% CI does not include 1.00.

**Table 2:** Hazard Ratio* (HR) and 95% confidence interval (95% CI) of fatal injuries among children 0-18 years old with foreign background by parental education, 1961-2007

Attained Parental Education	Male		Female		All	
	Event	HR (95% CI)	Event	HR (95% CI)	Event	HR (95% CI)
Unknown	5	**3.46 (1.45-8.53)**	5	**5.74 (2.29-14.40)**	10	**4.30 (2.26-8.17)**
<9 years	123	**2.22 (1.70-2.89)**	35	1.12 (0.74-1.69)	158	**1.81 (1.46-2.26)**
10-12 years	244	**1.60 (1.28-2.01)**	118	1.28 (0.95-1.74)	362	**1.48 (1.24-1.78)**
>=13 years	108	1(reference)	67	1(reference)	175	1(reference)

*Adjusted for attained age and calendar period of birth. Bold type, 95% CI does not include 1.00.

As shown in Table 1, risk of hospitalization due to unintentional injuries was significantly higher among children with lowest parental education (i.e., <9 years education) compared to those with highest parental education (i.e., >= 13 years education). We found similar results when stratifying results by sex, however, the risk was marginally significant among female children. 

When stratified results by external causes of injury (transportation-related injuries, drowning and suffocation, poisoning, fall, burns and fire, and other injuries), we found similar pattern; higher risk of hospitalization due to unintentional injuries among children with lowest parental education compared to those with highest parental education regardless of sex. However, there were few exceptions; no significant difference for injuries by drowning and suffocation and lower risk of hospitalization due to fall injuries among female children with lowest parental education compared to those with highest parental education (Table 3).

**Table 3:** Hazard Ratio* (HR) and 95% confidence interval (95% CI) of non-fatal injuries among children 0-18 years old with foreign background by parental education and external causes of injury, 1961-2007

Attained Parental Education	Male		Female		All	
	Event	HR (95% CI)	Event	HR (95% CI)	Event	HR (95% CI)
Transportation-related injuries								
Unknown	60	**1.51 (1.17-1.95)**	27	1.00 (0.68-1.46)	87	**1.30 (1.05-1.60)**
<9 years	1835	**1.31 (1.23-1.39)**	950	**1.12 (1.03-1.20)**	2785	**1.23 (1.18-1.29)**
10-12 years	5405	**1.28 (1.23-1.34)**	3210	**1.18 (1.12-1.25)**	8615	**1.24 (1.20-1.28)**
>=13 years	3131	1(reference)	2093	1(reference)	5224	1(reference)
Drowning and suffocation								
Unknown	11	0.69 (0.38-1.25)	10	1.05 (0.56-1.97)	21	0.82 (0.53-1.27)
<9 years	210	1.04 (0.89-1.22)	145	1.19 (0.98-1.45)	355	1.10 (0.97-1.24)
10-12 years	695	1.00 (0.90-1.12)	434	1.01 (0.88-1.16)	1129	1.00 (0.92-1.10)
>=13 years	573	1(reference)	364	1(reference)	937	1(reference)
Poisoning								
Unknown	24	0.70 (0.47-1.05)	31	1.28 (0.89-1.83)	55	0.94 (0.72-1.23)
<9 years	550	**1.21 (1.10-1.34)**	439	**1.15 (1.03-1.29)**	989	**1.18 (1.10-1.28)**
10-12 years	1809	**1.18 (1.10-1.27)**	1501	**1.23 (1.13-1.33)**	3310	**1.20 (1.14-1.27)**
>=13 years	1262	1(reference)	978	1(reference)	2240	1(reference)
Fall								
Unknown	127	0.97 (0.82-1.16)	90	0.97 (0.79-1.20)	217	0.97 (0.85-1.11)
<9 years	3247	1.00 (0.96-1.05)	1964	**0.93 (0.88-0.98)**	5211	0.98 (0.94-1.01)
10-12 years	11713	**1.05 (1.02-1.08)**	7485	**1.04 (1.00-1.07)**	19198	**1.05 (1.02-1.07)**
>=13 years	9183	1(reference)	6029	1(reference)	15212	1(reference)
Burns and fire								
Unknown	10	**2.10 (1.12-3.93)**	2	0.79 (0.20-3.18)	12	1.64 (0.93-2.91)
<9 years	194	**1.69 (1.42-2.00)**	104	**1.82 (1.44-2.29)**	298	**1.73 (1.51-1.99)**
10-12 years	534	**1.18 (1.04-1.34)**	313	**1.28 (1.09-1.51)**	847	**1.22 (1.10-1.35)**
>=13 years	453	1(reference)	264	1(reference)	717	1(reference)
Other injuries								
Unknown	64	1.16 (0.90-1.48)	33	0.96 (0.68-1.36)	97	1.08 (0.88-1.32)
<9 years	1666	**1.21 (1.14-1.29)**	823	1.05 (0.96-1.14)	2489	**1.15 (1.10-1.21)**
10-12 years	4990	**1.13 (1.08-1.18)**	2847	**1.08 (1.02-1.14)**	7837	**1.11 (1.07-1.15)**
>=13 years	3521	1(reference)	2175	1(reference)	5696	1(reference)

*Adjusted for attained age and calendar period of birth. Bold type, 95% CI does not include 1.00.

In respect to risk of fatal unintentional injuries, we encounter with lack of power because of few number of outcomes in most groups when stratified results by external causes of injury. However, when we had enough statistical power (transportation-related injuries, drowning and suffocation and burns and fire) we found higher risk of death due to unintentional injuries among children with lowest parental education compared to those with highest parental education that were mostly confined to male children(Table 4).

**Table 4:** Hazard Ratio* (HR) and 95% confidence interval (95% CI) of fatal injuries among children 0-18 years old with foreign background by parental education and external causes of injury, 1961-2007

Attained Parental Education	Male		Female		All	
	Event	HR (95% CI)	Event	HR (95% CI)	Event	HR (95% CI)
Transportation-related injuries								
Unknown	3	**4.43 (1.38-14.22)**	4	**8.52 (2.99-24.23)**	7	**6.11 (2.82-13.25)**
<9 years	72	**2.17 (1.53-3.07)**	22	1.16 (0.68-2.00)	94	**1.80 (1.35-2.41)**
10-12 years	148	**1.68 (1.24-2.27)**	76	1.46 (0.98-2.18)	224	**1.60 (1.26-2.03)**
>=13 years	60	1(reference)	36	1(reference)	96	1(reference)
Drowning and suffocation								
Unknown	2	3.46 (0.81-14.73)	0	N/A	2	2.99 (0.71-12.62)
<9 years	25	**1.85 (1.06-3.24)**	2	1.13 (0.20-6.35)	27	**1.78 (1.05-3.03)**
10-12 years	46	1.25 (0.77-2.02)	15	2.91 (0.96-8.84)	61	1.46 (0.94-2.25)
>=13 years	27	1(reference)	4	1(reference)	31	1(reference)
Poisoning								
Unknown	0	N/A	0	N/A	0	N/A
<9 years	1	2.92 (0.18-47.06)	0	N/A	1	1.02 (0.10-9.81)
10-12 years	9	7.05 (0.89-55.74)	3	1.22 (0.20-7.31)	12	3.16 (0.89-11.23)
>=13 years	1	1(reference)	2	1(reference)	3	1(reference)
Fall								
Unknown	0	N/A	0	N/A	0	N/A
<9 years	1	1.11 (0.10-12.81)	3	3.03 (0.49-18.76)	4	2.14 (0.52-8.82)
10-12 years	7	2.71 (0.56-13.16)	5	1.77 (0.34-9.19)	12	2.23 (0.72-6.95
>=13 years	2	1(reference)	2	1(reference)	4	1(reference)
Burns and fire								
Unknown	0	N/A	1	**11.73 (1.37-100.24)**	1	**9.47 (1.18-76.13)**
<9 years	9	**13.24 (2.81-62.35)**	5	1.72 (0.56-5.23)	14	**3.83 (1.71-8.58)**
10-12 years	12	**4.95 (1.10-22.17)**	10	0.92 (0.37-2.28)	22	1.65 (0.80-3.41)
>=13 years	2	1(reference)	9	1(reference)	11	1(reference)
Other injuries								
Unknown	0	N/A	0	N/A	0	N/A
<9 years	15	1.96 (0.95-4.05)	3	0.54 (0.15-1.93)	18	1.33 (0.73-2.44)
10-12 years	22	1.01 (0.53-1.93)	9	0.50 (0.22-1.17)	31	0.78 (0.47-1.29)
>=13 years	16	1(reference)	14	1(reference)	30	1(reference)

*Adjusted for attained age and calendar period of birth. Bold type, 95% CI does not include 1.00.

## Discussion

In general, we found significantly higher risk of non-fatal and fatal unintentional injuries among foreign-background children with lowest parental education compared to that among children with highest parental education after adjustment for age and calendar period of birth. 

It should be emphasized that injuries are caused by a range of intra- and inter-personal and environmental factors, and no single factor can explain differences found among children of foreign-background. There are epidemiological evidences, however, that introduced a number of variables responsible for increased risk for injury among children. ^21,22^ Because of certain socio-cultural factors, foreign-born persons generally have more favorable behavioral familial, and social support factors such as family networks, relationships within families and the interaction between recent immigrant families and earlier immigrants of the same ethnic group.^23^ However, we encounter with a very heterogenic group of people and this means we need more knowledge on injury risk among immigrants to perform injury prevention.^24,25^

Low childhood mortality is considered to be good indicators of public health in a society and its success in implementation of general population’s welfare, living conditions, and safety. Sweden considered injury as a public health problem and the Joint Committee for the Prevention of Accidents to Children was set up in 1950s and now the Swedish Rescue Services Agency is responsible for the National Safety Promotion Program. This ended up with a general decrease in road and other unintentional injuries.^26^ The main explanations for the declining mortality and morbidity rates are probably better social and social conditions and the application of a national strategy for safety promotion. The mentioned strategy has taken improvements in infrastructure, child restraint systems, helmet use, swimming competence, use of life vests in recreational activities, and availability of public child health care for all.^9^

The major strengths of our study are the nationwide design with a long follow-up of all foreign-born children during the study period. We used Total Population Register as the source for country of birth in our study. Thus, there is virtually no concern about consistency in definition of migrant status in our studies. We had enough statistical power to show with statistical significance likely available differences for fatal and non-fatal unintentional injuries and their subcategories, sex groups, and countries of origin.

One of the important weaknesses of our study is that the information was limited to only registers. Thus, we were not able to consider behaviors, attitudes, language skills, and other factors, which may be important for injury risk.

## Conclusion

Although the results of our analysis cannot be directly generalized to other countries, however, similar patterns may be seen. In addition, our study was able to add extra evidences indicating that the priority areas identified in other studies is in line with earlier studies and should be considered for further attention. As even one child death is of importance, preventive actions should direct towards children in immigrant families with low parental education. Additional research is needed to establish the specific determinants for the increased mortality among ethnic groups and to identify ways to effectively tackle them.
